# Editorial: Short stature: beyond growth hormone

**DOI:** 10.3389/fendo.2024.1403112

**Published:** 2024-03-28

**Authors:** Lukas Plachy, Annalisa Deodati, Gianluca Tornese

**Affiliations:** ^1^ Department of Pediatrics, Second Faculty of Medicine, Charles University in Prague and Motol University Hospital, Prague, Czechia; ^2^ Endocrinology and Diabetes Unit, “Bambino Gesù” Children’s Hospital, Rome, Italy; ^3^ Department of Systems Medicine, University of Rome Tor Vergata, Rome, Italy; ^4^ Department of Medicine, Surgery and Health Sciences, University of Trieste, Trieste, Italy; ^5^ Institute for Maternal and Child Health IRCCS “Burlo Garofolo”, Trieste, Italy

**Keywords:** endocrinological diseases, stimulation tests, growth hormone, genetics, short stature

Short stature is the most common cause of referral to pediatric endocrinology units (1) and can be defined as a multifactorial condition regulated by genetic, epigenetic, and environmental factors (2). Traditionally, growth hormone (GH) has been considered the main regulator of growth. However, as understanding of short stature pathogenesis advances, a new concept has been proposed: the role of GH in the regulation of growth is only one of many factors influencing growth plate physiology (3). Moreover, the diagnostic methods currently used to diagnose GH deficiency (GHD) are known to have low specificity, leading to frequent false positive results (4). Children with diagnosed GHD are therefore believed to have variable etiology of their growth disorder, frequently independent of GH secretion (5).

One of the major topics in current pediatric endocrinology is whether it is possible to improve the poor accuracy of GH stimulation tests. One possibility might be the optimization of sex-steroid priming (6). Partepone et al. presented a comprehensive review regarding this topic. The authors highlighted a close link between sex steroids and GH secretion leading to a higher probability of false positive results in children with delayed onset of puberty and consequent GH overtreatment. The same mechanism, on the other hand, may lead to a non-physiological GH peak, resulting in missing the diagnosis in children with real GH deficiency (GHD) in case sex-steroid priming is performed. So far, there is no agreement Regarding the indication and management of sex-steroid priming. Another issue that might lead to an inaccurate diagnosis of GHD is bone age (BA) evaluation. Delayed BA is mandatory before making the GHD diagnosis in some countries (7), however, the subjective nature of the evaluation is considered its main disadvantage. Maratova et al. evaluated an automated software for BA evaluation and proved its good accuracy.

A lasting controversy in the current way to diagnose GHD was supported by Plachy et al. Using next-generation sequencing methods, the authors genetically examined children with isolated growth hormone deficiency (GHD) and familial short stature. Interestingly, the genetic results frequently did not correspond with the previous diagnosis of GHD – 67% of children with a clinical diagnosis of GHD and a genetic etiology of short stature had proven primary growth plate disorder. Another point of view on the same topic was presented by Lanzetta et al. In their retrospective analysis of children with a clinical and laboratory diagnosis of GHD, they compared children with or without an identifiable genetic, functional, or anatomical cause of GHD, namely definite GHD or short stature unresponsive to stimulation tests (SUS). These two groups differed significantly in pretreatment IGF-1 concentration and their increase after GH treatment initiation, in prevalence of pathological retesting, and of being overweight/obese at the end of treatment. However, the response to GH treatment in terms of near-adult height did not differ between the groups. Despite lasting doubts regarding the accuracy of GHD diagnostics, children diagnosed with “GHD” might profit from GH therapy even when another etiology of short stature is suspected.

The etiology of short stature other than GHD was covered by two other articles in our Research Topic. Mastromauro et al. wrote a review presenting growth hormone insensitivity (GHI) as a broad spectrum of disorders with a variable clinical picture. Since Laron described homozygous mutations in the gene for the GH receptor as the first mechanism causing GHI, many novel causes of GHI have been described, demonstrating the complexity of GHI and its role in the growth regulation. Another numerous and etiologically highly variable group of children are those born small for gestational age (SGA) with persistent short stature (8). In a retrospective study, Becker et al. compared clinical features and responses to GH treatment of SGA children with and without syndromic signs. They discovered that syndromic SGA children were shorter at the initiation of GH treatment, started GH therapy earlier, and reached a shorter adult height despite receiving higher doses of GH.

The etiology of growth disorders is, therefore, more complex than originally expected and is not just a matter of hormones. To understand it better, we must think far beyond GH.

## Author contributions

LP: Writing – original draft, Writing – review & editing. AD: Writing – original draft, Writing – review & editing. GT: Writing – original draft, Writing – review & editing.
